# Genome-wide analysis in multiple-case families: assessing the relationship between triglyceride and methylation

**DOI:** 10.1186/s12919-018-0123-z

**Published:** 2018-09-17

**Authors:** Angga M. Fuady, Renaud L. M. Tissier, Jeanine J. Houwing-Duistermaat

**Affiliations:** 1 Department of Medical Statistics and Bioinformatics, Leiden University Medical Center, Einthovenweg 20, 2333 ZC Leiden, The Netherlands; 2 Department of Statistics, University of Leeds, Leeds, LS2 9JT UK

## Abstract

The main goal of this paper is to estimate the effect of triglyceride levels on methylation of cytosine-phosphate-guanine (CpG) sites in multiple-case families. These families are selected because they have 2 or more cases of metabolic syndrome (primary phenotype). The methylations at the CpG sites are the secondary phenotypes. Ascertainment corrections are needed when there is an association between the primary and secondary phenotype. We will apply the newly developed secondary phenotype analysis for multiple-case family studies to identify CpG sites where methylations are influenced by triglyceride levels. Our second goal is to compare the performance of the naïve approach, which ignores the sampling of the families, SOLAR (Sequential Oligogenic Linkage Analysis Routines), which adjusts for ascertainment via probands, and the secondary phenotype approach. The analysis of possible CpG sites associated with triglyceride levels shows results consistent with the literature when using the secondary phenotype approach. Overall, the secondary phenotype approach performed well, but the comparison of the different approaches does not show significant differences between them. However, for genome-wide applications, we recommend using the secondary phenotype approach when there is an association between primary and secondary phenotypes, and to use the naïve approach otherwise.

## Background

The multiple-case family design oversamples families with cases of metabolic syndrome (primary phenotype). To obtain unbiased estimates of the model parameters, adjustments for this oversampling are required. Some work has been done when the primary phenotype is modeled [1]. However, it is often overlooked that ascertainment corrections are also needed when a secondary phenotype is modeled and the primary and secondary phenotype are correlated. A combination of the retrospective likelihood and a joint model for the primary and secondary phenotypes appears to provide unbiased estimates of the model parameters [2, 3]. Unfortunately for family-based data sets, the within-family correlation also must be modeled, which yields integration over a multivariate distribution of a dimension of twice the size of the family. As a consequence of computations of high-dimensional integrals in large pedigrees, model fitting becomes time-consuming and applications in genome-wide settings are almost infeasible.

**Fig. 1:**
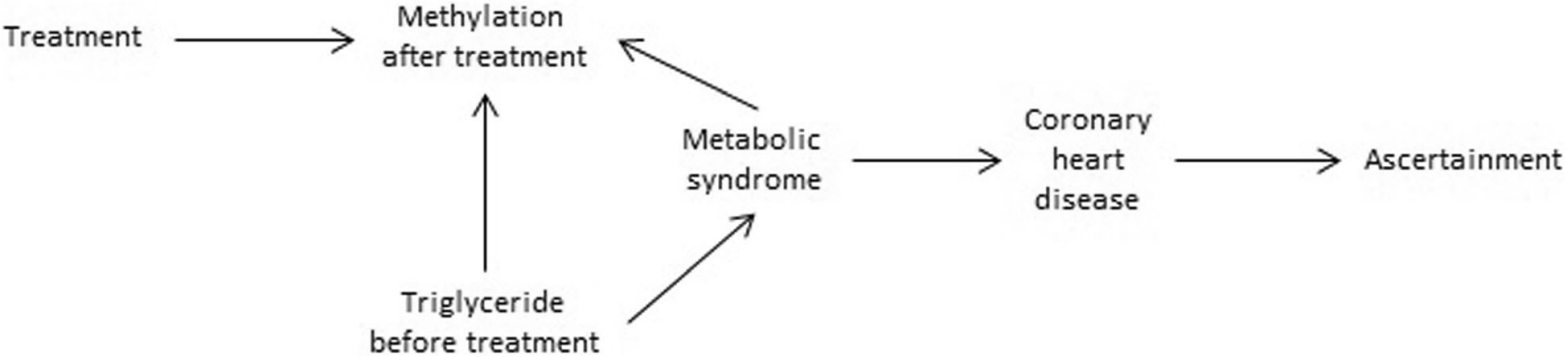
Directed acyclic graph illustrating the existing association between each node

Our main goal is to investigate the total effect of triglyceride (TG) levels before treatment on methylation after treatment in families with coronary heart disease (CHD). We do not have information on CHD, but we have information on metabolic syndrome (MetS), which is known to influence CHD [4]. Note that TG level is associated with MetS because of 1 diagnosis criteria of MetS (fasting blood TG level ≥ 150 mg/dL) [5]. Fig. 1 shows the relationship between the variables of interest in a directed acyclic graph [2, 6]. In this directed acyclic graph, the effect of TGs on methylation of a cytosine-phosphate-guanine (CpG) site might be either direct or via the mediators MetS and TG at follow-up. To assess the total effect of TG level at baseline on methylation after treatment, we assume that there are no confounders for the relationship between TG level at baseline and methylation after treatment. The variable *S* represents the ascertainment process. We assume that *S* is solely based on CHD. We assume that there are no confounders for the relationship between CHD and MetS and that the TG level has no direct effect at baseline on CHD.

Our second goal is to compare the performance of various methods for ascertainment correction in the multiple-case family design. Although the secondary phenotype approach provides unbiased estimates, computations are time-consuming. A second often used method is available in SOLAR (Sequential Oligogenic Linkage Analysis Routines) [7]. This method provides valid estimates for secondary phenotype analysis in the proband family design, where family members of probands (typically cases, but controls also might be considered) are recruited. Ascertainment correction for this design involves conditioning the likelihood function on the outcome of the proband. Computations are fast and analyses at genome-wide scale are hence feasible. We investigate whether we can apply this method to the multiple-case families of GAW20 by assigning the proband status to individuals. Finally, we consider the naïve approach, which does not take the family design into account.

## Methods

### Study sample

We analyze the family data from the Genetics of Lipid Lowering Drugs and Diet Network (GOLDN) study [8]. We restrict ourselves to 530 participants who have a complete measurement of TG levels for time points 1 and 2 and of methylation at CpG sites for time point 4 (after treatment). We have data on more than 450,000 CpG sites covering all autosomal chromosomes. The families of the GOLDN study are a subsample of the Family Heart Study of two centers: Minneapolis, MN, and Salt Lake City, UT. In the Family Heart Study, families with at least 2 CHD cases and a family risk score of 0.5 or higher were eligible [9]. Because we do not have information about CHD cases in the families, we used the related outcome, MetS, as the primary phenotype.

### Secondary phenotype approach

To assess the effect of TG level on methylation at a CpG site (secondary phenotype), we used the newly developed secondary phenotype approach [3], which fits a joint model to the secondary phenotype (methylation) and the primary phenotype (MetS). The retrospective likelihood that conditions on MetS is used. If the ascertainment *S* only depends on the primary phenotype MetS, this method corrects for the ascertainment [3]. The retrospective likelihood for modeling the effect of TG on methylation is:

1 $$ P\left(M| TG, MS\right)=\frac{P\left( MS,M\ \right| TG\Big)\ P(TG)}{P\left( MS| TG\right)P(TG)}=\frac{P\left( MS,M\ \right| TG\Big)\ }{P\left( MS| TG\right)} $$

where *MS* = MetS and *M* = methylation. For the numerator, we use a probit link to model the conditional joint distribution of the binary variable *MS* and the continuous variable *M* in families given *TG*. Let *MS*^′^ be the normally distributed latent variable for *MS*, that is, the liability score. The conditional joint density *P*(*MS* = 1, *M* = *m*| *TG*) can be obtained from the joint distribution *P*(*MS*^′^, *M*| *TG*) from the following formula:

$$ P\left( MS=1,M=m| TG\right)=\underset{0}{\overset{\infty }{\int }}P\left(m{s}^{\prime },M=m| TG\right){dms}^{\prime } $$

where *m* and *ms*^′^ are the realization of *M* and *MS*^′^, respectively. Furthermore, *P*(*MS* = 0, *M* = *m*| *TG*) = 1 − *P*(*MS* = 1, *M* = *m*| *TG*).For the distribution of *MS′* and *M* given *TG* we use a multivariate Gaussian distribution with means *μ*_*MS*′_ + *β*_*MS*′_*TG* and *μ*_*M*_ + *β*_*M*_*TG*, respectively, and variance–covariance matrix $$ \Sigma =\left(\begin{array}{cc}{\varSigma}_{MS\prime }& {\varSigma}_{M\  MS\prime}\\ {}{\varSigma}_{MS\prime M}& {\varSigma}_M\end{array}\right), $$ where the covariance structures are modeled with normally distributed zero mean random effects as follows: *Σ*_*MS*′_ =  *var* (*g*_*MS*′_ + *u* + *ϵ*_*MS*_), *Σ*_*M*_ =  *var* (*g*_*M*_ + *u* + *ϵ*_*M*_);that is, the covariance of *MS* and *M* within the family, and ${\varSigma}_{M\  MS\prime }=\mathit{\operatorname{cov}}\left({g}_M+u+{\epsilon}_M,{g}_{MS^{\prime }}+u+{\epsilon}_{MS\prime}\right)$ the covariance of the random effects modeling the correlation between the two outcomes within the family. Here *g*_*MS*′_ *g*_*M*_ are the genetic random effects with correlation structure given by the kinship coefficient; *ϵ*_*M*_ is the residual; and *u* is the shared random effects between primary and secondary phenotype. For identifiability reasons of the variance parameters of *MS*^′^, *ϵ*_*MS*′_ is fixed to 1. Finally, the denominator in eq. (1), *P*(*MS*| *TG*), can be obtained by integrating *P*(*MS*, *M*| *TG*) over the distribution of *M*. See Tissier et al. [3] for details.

### Naïve approach

For each CpG site, a linear mixed-effect model is used to assess the effect of TG level on methylation. The correlation within the families is modeled by polygenic effects *g*_*M*_. The *lmekin* function from package *coxme* in R is used to fit these models. This approach provides unbiased results when there is no association between the primary and secondary phenotype.

### Adjusting for selection based on proband

A second approach is to assign a proband status to some of the family members and to use methods for the proband family design for data analysis. We considered 2 sets of probands, namely a proband status which is equivalent to having MetS (SOLAR [MetS]) and to being the oldest member of the family (SOLAR [OLD]). Proband status based on MetS was chosen, because families were selected based on CHD and MetS is associated with CHD [10]. Using the oldest member of a family as proband is motivated by fact that recruitment of the families was conducted decades ago. The CHD cases that satisfy the selection criteria are likely to be deceased and the oldest family member might be the link between these CHD cases and the current family members. We used the function polygenic as implemented in SOLAR (Texas Biomedical Research Institute, San Antonio, TX) [7].

Corrections for ascertainment were made by conditioning the likelihood for each pedigree on the trait values of the pedigree probands [11]. Let *I*^*P*^ be the indicator for the proband in the family, which represents the outcomes of the proband (ie, *M*^*P*^ and *TG*^*P*^), *i* the index of the family, ${M}_i^F$ and $T{G}_i^F$ the vectors of the outcomes for the whole family, and *S* the ascertainment process. For a family *i*, the likelihood used by SOLAR can be written as:

$$ P\left({M}_i^F\right|T{G}_i^F,{S}_i\Big)=\frac{P\left({M}_i^F\right|T{G}_i^F\Big)}{P\left({M}_i^P\right|T{G}_i^P\Big)} $$

The SOLAR approach is based on 2 assumptions. The first assumption is that the ascertainment is solely based on the outcomes of the probands:

$$ P\left({M}_i^F\right|T{G}_i^F,{S}_i\left)=P\left({M}_i^F\right|T{G}_i^F,{I}^P\right)=P\left({M}_i^F\right|{TG}_i^F,T{G}_i^P,{M}_i^P\Big) $$

By use of the Bayes rule we obtain:

$$ P\left({M}_i^F\right|{TG}_i^F,T{G}_i^P,{M}_i^P\Big)=\frac{P\left({M}_i^F,{M}_i^P,T{G}_i^F,T{G}_i^P\right)}{P\left({M}_i^P,T{G}_i^F,T{G}_i^P\right)}=\frac{P\left({M}_i^F,{M}_i^P\right|T{G}_i^F,T{G}_i^P\Big)}{P\left({M}_i^P\right|T{G}_i^F,T{G}_i^P\Big)} $$

The second assumption made is that the outcome of the proband does not depend on the covariate values of the family members given its own covariate value. Therefore, $P\left({M}_i^P\right|T{G}_i^F,T{G}_i^P\left)=P\left({M}_i^P\right|T{G}_i^P\right)$. Finally, as ${M}_i^P\subset {M}_i^F$ and $T{G}_i^P\subset T{G}_i^F,$ the numerator becomes $P\left({M}_i^F\right|T{G}_i^F\Big)$.

The log-likelihood maximized by SOLAR is then:

$$ L=\sum \limits_i\mathit{\ln}\left(P\left({M}_i^F\right|T{G}_i^F\right)\Big)-\mathit{\ln}\left(P\left({M}_i^P|T{G}_i^P\right)\right) $$

A family-wise error rate of 0.05 is used throughout this article and we apply a Bonferroni correction to obtain the per-test *p* value.

### Strategy

Because it is not feasible to apply the secondary phenotype approach to all CpG sites, we use a 2-step approach to estimate the effect of TG level on methylation. First, we test for each CpG site whether the primary phenotype MetS has an effect on methylation using a linear mixed model to take into account the existing correlation among family members. For this analysis, there is no need to adjust for ascertainment, as we assume that ascertainment is via MetS. From these analyses, we identify the group of CpG sites that are significantly influenced by MetS (*p* < 1.1e-7). Second, we estimate the effect of TG level on methylation for each CpG site. For the group of CpG sites with methylation significantly influenced by MetS, we apply the secondary phenotype approach. For the remaining CpG sites, we apply the naïve approach.

To study the performance of SOLAR(MetS), SOLAR(OLD), the secondary phenotype, and naïve approaches, we apply these approaches to a set of 1000 randomly selected CpG sites for which MetS has no influence on methylation (test specific *p*>0.8). We computed the average over the 1000 CpG sites of the square differences between the estimates of the effect of TG level on methylation obtained by the approaches used to adjust for ascertainment and the naïve approach:

$$ av{g}_{diff}=\frac{\sum {\left({\beta}_1-{\beta}_2\right)}^2}{1000} $$

*β*_1_ and *β*_2_ are the estimates of the effect of TG level on methylation from either secondary phenotype or SOLAR and naïve approaches, respectively. The estimates of the parameters represent the increase in methylation per unit of TG in dL/mg. We apply the delta method, where we use the estimated correlation between the parameters over all CpG sites, to obtain the standard errors. Finally, we assess the performance of the Wald statistics corresponding to parameter estimates of the 4 approaches in the 1000 randomly selected CpG sites for which MetS has no influence on methylation by a quantile–quantile (Q-Q) plot of the *p* values.

## Results

We applied the 2-step approach to 463,995 CpG sites located at the autosomal chromosomes. After the first step of the analysis, 3842 CpG sites appear to be significantly influenced by MetS (*p* < 1.1e-7). For these CpG sites, the secondary phenotype approach was used to assess the effect of TG level on methylation. Methylation at the remaining CpG sites were analyzed using the naïve approach. Table 1 shows the results. The secondary phenotype approach identified 294 CpG sites as associated with TG level in the group of CpG sites previously found to be associated with MetS. One of the identified CpG sites is cg00574958, which has a *p* value of 6.7e-10. This CpG site was identified as being associated with TG level by Irvin et al. [8].

**Table 1:** The number of significant CpG sites using the 2-step approach with MetS as the primary phenotype

CpG sites (*n* = 463,995)	Approaches	Number associated with TG level
Associated with MetS (3842)	Secondary phenotype	294 (7.6%)
Not associated with MetS (460,153)	Naïve	0

**Fig. 2:**
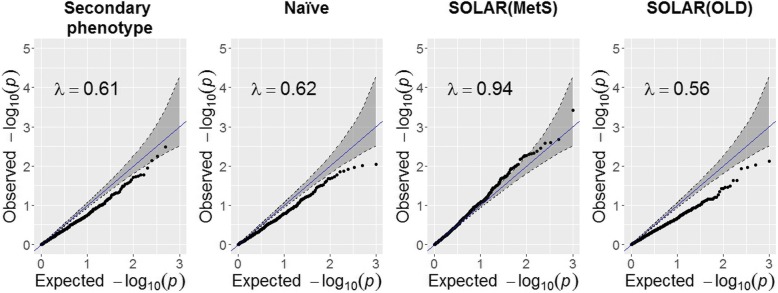
Q-Q plots of *p* values corresponding to the Wald statistic of 1000 randomly chosen CpG sites in which methylation is not influenced by MetS

Regarding the comparison of the different ascertainment correction approaches, Table 2 shows the average of the square differences between the estimates of the parameters representing the effect of TG level on methylation obtained from the approach that attempts to adjust for ascertainment and the naïve approach. For the group of CpG sites associated with MetS, the average of the square difference to naïve approach is 0.120 for secondary phenotype, 0.223 for SOLAR(MetS), and 0.006 for SOLAR(OLD). The average square difference in the group of CpG sites that are not associated with MetS using the naïve approach was not significantly different from 0 except for SOLAR(OLD). Finally, Fig. 2 shows the Q-Q plots of the *p* values of the Wald tests for the 1000 randomly selected not associated CpG sites for the 4 approaches. The secondary phenotype approach and the naïve approach are a slightly conservative whereas SOLAR(MetS) is too liberal. SOLAR(OLD) is quite conservative.

**Table 2:** Average of the square of the differences between the effect estimates between 2 approaches (in brackets are the 95% confidence intervals)

Group of CpG with methylation	Naïve and secondary phenotype	Naïve and SOLAR(MetS)	Naïve and SOLAR(OLD)
Associated with MetS	0.120 [−0.115, 0.355]	0.223 [−0.208, 0.654]	0.006 [−0.001, 0.013]
Not associated with MetS	0.001 [−0.002, 0.004]	0.050 [−0.016, 0.116]	0.007 [0.005, 0.008]

## Discussion and conclusions

We studied the performance of the secondary phenotype approach and SOLAR with 2 proband definitions. When there is no association between the primary and the secondary phenotype, the naïve and the secondary phenotype approach provide similar results [3]. In contrast, the estimated association effects from SOLAR(MetS) and SOLAR(OLD) in this group are not similar to the ones obtained by the naïve approach. The Q-Q plots show that the secondary and the naïve approach perform similarly and are a slightly conservative in terms of distribution of the test statistic under the null hypothesis. SOLAR appears to be either conservative or too liberal for these data. The method of SOLAR conditions on the proband outcome, which might not provide valid estimators if the outcome of the proband is a collider. Furthermore, SOLAR assumes that the outcome of the proband does not depend on the covariate values of the family members given its own covariate value, which might not be correct in the presence of unobserved confounders. Moreover, for the selection scheme used in this study, the secondary phenotype approach is most appropriate. However, in contrast to the secondary phenotype approach, SOLAR can be applied in genome-wide settings.

The directed acyclic graph clarifies the assumptions that are made in our analysis. The assumptions that TG level has no direct effect on CHD and that there are no unobserved confounders for MetS and CHD might not be valid. Indeed, Holmes et al. [12] showed a causal effect of TG level on CHD using Mendelian randomization. However, these authors do not consider MetS; consequently, MetS might be in the path between TG level and CHD and our model cannot be excluded. Moreover, Mendelian randomization also makes many assumptions [13]. Furthermore, our result that the secondary phenotype approach gives estimates similar to those given by the naïve approach when there is no relationship between primary (MetS) and secondary (methylation) suggests that our directed acyclic graph is appropriate.

Because the secondary phenotype approach is known to be robust, we advocate using the secondary phenotype approach for CpG sites that are significantly associated with the primary outcome, MetS.
